# Regulatory flexibility and school climate moderate the relationship between stress exposure and depression severity in school educators

**DOI:** 10.1038/s41598-024-62150-8

**Published:** 2024-05-21

**Authors:** Hagit Nizri, Alla Hemi, Einat Levy-Gigi

**Affiliations:** 1 Faculty of Education, Bar Ilan University, Ramat Gan, Israel; 2 The Multidisciplinary Brain Research Center, Bar Ilan University, Ramat Gan, Israel

**Keywords:** Stress exposure, Depression, School climate, Regulatory flexibility, Educators, Health occupations, Signs and symptoms, Therapeutics

## Abstract

School-related stress may impair the mental health and the ability of educators to function at school adaptively. According to the Conservation of Resources (COR) model, coping with stress is affected by internal personal resources and external interpersonal resources. The current study focused on regulatory flexibility as an internal personal resource and school climate as an external interpersonal resource. It tested their moderating role in the relationship between school-related stress exposure and depressive symptoms. 1530 educators participated in the study. The results revealed that school climate and regulatory flexibility play a significant role in determining the severity of depressive symptoms following stress exposure. Specifically, when either school climate and/or regulatory flexibility were low, there was a positive association between school-related stress exposure and depressive symptoms. Hence, greater exposure was associated with increased depressive symptoms. However, when both school climate and regulatory flexibility were higher, there were no associations between stress exposure and symptoms. Therefore, these educators showed significantly lower depressive symptoms independent of their stress exposure. The findings shed light on the importance of both internal and external resources in reducing the aversive effects of school-related stress. The study may pave the way to developing tailored interventions to reduce depressive symptoms and enhance well-being in educators.

## Introduction

Educators frequently encounter stressful situations inherent to their profession, including exposure to various forms of violence (e.g., verbal or physical incidents among students or directed at staff) and managing student misbehaviors^1^. Additionally, they contend with stressors arising from a demanding workload and from the emotional support they provide to students facing various challenges like familial loss or illness^2,3^. The current study builds on the conservation of resources (COR) model^4–6^ to test how personal and interpersonal resources affect the ability of educators to cope with such stressors adaptively.

Previous studies have shown that exposure to school-related stress was associated with decreased mental health and greater depression severity in educators^7–14^. Specifically, educators with greater perceived stress reported reduced emotional well-being^15–18^. In addition, educators who had to deal with greater disruptive behaviors, poorer student motivation, and increased workload reported higher distress and depressive symptoms^19,20^. Such symptoms may reduce educators' general functioning^12,13,20^ and impair their work-related satisfaction and motivation^14,19,21^. The relationship between school-related stress and the tendency to develop depressive symptoms, together with the significant negative impact of depression on educators, make it an important target for investigation.

According to the conservation of resources (COR) model^5,6,22^ the impact of stressful events depends on the availability of various resources^14,23–25^. Specifically, individuals with greater resources are less vulnerable to loss and more capable of gaining resources. On the other hand, those with fewer resources are more prone to loss and less able to gain resources and, therefore, are more vulnerable to conditions of continuous stress and/or repeated traumatic exposure^26–30^. Moreover, the COR model claims that initial resource loss leads to future losses^31,32^. This may suggest a vicious cycle in which limited resources under conditions of continual exposure to stress lead to further resource depletion^33^.

The model distinguishes between two types of resources: internal personal resources (e.g., self-esteem, mastery, flexibility) and external interpersonal resources (e.g., school climate, availability of materials, social support). In line with the COR model, the current study tested the effect of two possible resources: flexibility, which represents an internal personal resource, and school climate, which represents an external interpersonal resource, on the tendency of educators to develop depressive symptoms following exposure to stressful incidents. 

*Flexibility* is the ability to change behaviors and thoughts in accordance with the changing circumstances. It is considered to have significant mental health implications and contribute to the development of resilience^34,35^. Whereas early studies refer to flexibility as a broad concept^36^, recent studies propose that various types of flexibility may have different effects^37,38^. The current study focused on regulatory flexibility, which is the ability to modulate emotional experiences and choose regulatory strategies that suit situational demands^37,39^. Since this is a relatively new concept, to the best of our knowledge, studies have yet to test its effects on the well-being of educators. A relatively small number of studies have focused on the effects of other aspects of flexibility. For example, it was found that cognitive flexibility was associated with better coping with school-related stressors^40^ and positively predicted psychological hardiness^41^. In addition, it was found that interventions aiming at improving psychological flexibility contribute to the general well-being of teachers^42^.

Studies on first responders and the general population that examined regulatory flexibility have demonstrated a significant link between low levels of regulatory flexibility and elevated levels of depression^43,44^. Moreover, it was found that regulatory flexibility moderates the relationship between traumatic exposure and the tendency to develop PTSD symptoms among first responders. Specifically, for those with poor regulatory flexibility, greater traumatic exposure was associated with greater PTSD severity. In contrast, no such connection existed in those with high regulatory flexibility^37,38,44,45^. This may suggest that regulatory flexibility serves as a protective factor against the harmful effects of stress and trauma exposure across time and may also affect the way educators cope with school-related stress.

*School climate* reflects the school norms, values, interpersonal relationships, teaching and learning practices, and organizational structures and can be viewed as one of the external- interpersonal resources for educators^46,47^. Previous studies consistently demonstrated that a better school climate is associated with greater resilience and significantly fewer psychological problems^40,46,47^. Specifically, educators who work in schools with a better climate reported higher well-being than those who work in schools with a poor climate^46^. However, to date, no study has tested whether school climate moderates the relationship between levels of school-related stress and overall well-being or, specifically, depressive symptoms.

Based on the COR model and the existing findings, which show the beneficial effects of greater regulatory flexibility and better school climate, we hypothesized that educators with low regulatory flexibility and poor school climate would show a positive relationship between stress exposure and depression severity. Conversely, there would be no significant relationship between stress exposure and depression in educators with high levels of regulatory flexibility and a positive school climate.

## Methods

### Participants

Power analysis revealed the need for a sample size of 1361 per group to ensure 95% power at a 1% significance level. We enlarged the sample size by 15% to account for possible participant dropout, resulting in a sample size of 1530 participants. The school educators (86% female, *M*_age_ = 44.01, *SD* = 10.30) volunteered to participate in the study and completed it online. Table 1 displays the frequency and percentage of the sample's demographic characteristics. All the participants signed the consent form before the beginning of the experiment. All the research and the methods were conducted following the Declaration of Helsinki. The Ethics Committee of Bar-Ilan University approved the study. All the participants signed an informed consent form.

**Table 1:** The demographic characteristics of the sample presented by frequency and percentage.

Demographic variable	Frequency	Percent
Gender		
Male	208	13.59
Female	1322	86.41
Economic status		
Below average	92	6.00
Average	1042	68.10
Above average	379	24.80
Much above average	17	1.10
School Level		
Primary	655	42.81
Secondary	875	57.19
Workload		
Fulltime	1051	68.69
Parttime	479	31.31
Role		
Principal	91	5.95
Counsellor	104	6.80
Homeroom teacher	558	36.47
Pedagogic coordinator	203	13.27
Subject teacher	429	28.04
Support and administrative staff	155	10.13

## Measures

*Repeated exposure to school-related stress*^40,46,47^ is a 10-item questionnaire that assesses the frequency of exposure to common school-related stressful incidents in an average month. The respondents were asked to rate their exposure frequency to each incident on a scale from 1 (not at all) to 6 (at least a few times a week). The scale's internal consistency in the current study was *α* = 0.82.

*The Perceived Ability to Cope with Trauma* (PACT)^43^ is a 20-item questionnaire that measures the ability to cope and apply various regulatory strategies flexibly on a 7-point scale ranging from 1 (unable) to 7 (extremely able). The scale's internal consistency in the current study was *α* = 0.91.

*School climate* (The Counselor–Psychological Service at the Ministry of Education in Israel, *2021*) is a 33-statement questionnaire developed by the Ministry of Education and used to assess school climate across the country in the past three years. The questionnaire assesses how the educators perceive the school regarding the level of violence, sense of belonging and identification, and feeling of effective functioning. The agreement with each statement is rated on a 5-point Likert scale from 1 (disagree) to 5 (strongly agree). The internal consistency of the scale in the current study *α* = 0.91.

*The Beck Depression Inventory* (BDI-II)^48^ is a 21-item scale that assesses symptoms of depression over the past two weeks on a scale from 0 to 3. The internal consistency in the current study is *α* = 0.87.

## Results

### Zero-Order Correlations

The correlations between all study variables are presented in Table 2. In line with previous findings, school-related stress exposure positively correlated with depressive symptoms^13^ and negatively correlated with school climate^49^. In addition, as expected, depression negatively correlated with regulatory flexibility and school climate^50,51^. Finally, regulatory flexibility and school climate had a significant positive correlation. These results suggest that both the internal (high regulatory flexibility) and external (a positive school climate) resources are related and associated with reduced depression severity in school educators.

**Table 2:** Descriptive statistics, reliabilities, and correlations of all study variables.

Variable	M (SD)	*α*	Stress exposure	Regulatory flexibility	School Climate
Stress exposure	2.16 (0.82)	.80			
Regulatory flexibility	9.09 (2.00)	.91	.01		
School Climate	4.13 (0.48)	.91	− .09***	.28***	
Depression	5.84 (6.31)	.89	.18***	− .26***	− .33***

*** *p* < .001.

### Regulatory flexibility and school climate as moderators in the relationship between stress exposure and depression among educators

To test the hypothesis that regulatory flexibility and school climate moderate the relationship between school-related stress exposure and depression, we employed Hayes’s PROCESS macro (2017; model 2). In this model, school-related stress exposure, regulatory flexibility, school climate, and depression were treated as independent moderators and outcome variables, respectively. To control for possible gender effects, gender was included as a covariate. The results are summarized in Table 3. The general model was significant (*R*^2^ = 0.17, *F* (6, 1523) = 52.53, *p* < 0.001). Core analyses revealed a significant positive main effect of stress exposure on depression and significant negative main effects of regulatory flexibility and school climate on depression.

**Table 3:** Estimated coefficients, standard errors and 95% confidence intervals for independent and moderator variables in the model predicting levels of depression based on level of exposure moderated by trauma-related regulatory flexibility and school climate.

Predictor variables	*B*	*S.E*	*t*	*p*	95% *CI*	
					Low	High
Exposure	*1.1*2	0.18	6.18***	.000	0.76	1.47
*R*egulatory flexibility	− 0.59	0.08	− 7.62***	.000	− 0.74	− 0.44
*School climate*	− 3.37	*0.32*	− 10.41***	.000	− 4.01	− 2.74
*Exposure* × *Regulatory flexibility*	− 0.19	0.10	− 1.97*	.049	− 0.38	*0.00*
Exposure × School climate	− 0.98	0.36	− 2.75**	.006	− 1.68	− 0.28
*Gender*	*0.90*	*0.44*	*2.0*8*	.038	*0.0*5	*1.7*6

* p < .05, ** *p* < .01*,* *** *p* < .001.

The three-way interaction was significant, *F*(2,1523) = 6.87, *p* = .001. CI = Confidence interval.

**Figure 1:**
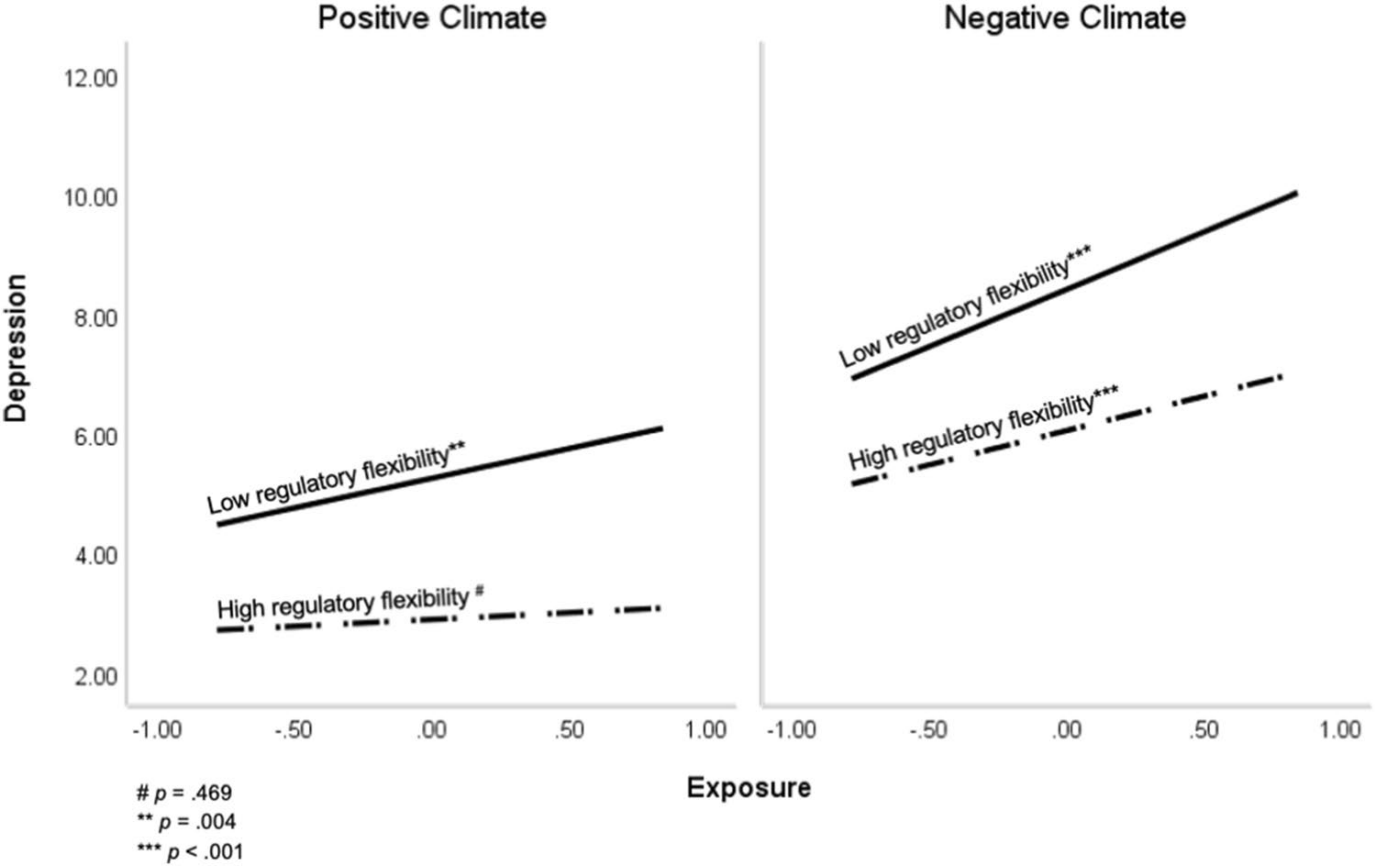
The effect of school-related stress exposure, regulatory flexibility, and school climate on depression severity in educators.

Most importantly, there was a significant three-way interaction of school-related stress exposure, regulatory flexibility, and school climate on the level of depression. To interpret the results, we conducted bootstrapping confidence intervals (95%) evaluating the magnitude of the relationship between stress exposure and depression severity for participants with **low** levels of regulatory flexibility and **low** levels of positive school climate (− −); **low** levels of regulatory flexibility and **high** levels of positive school climate (- +); **high** levels of regulatory flexibility and **low** levels of positive school climate (+ −) and **high** levels of regulatory flexibility and **high** levels of positive school climate (+ +) (See Fig. 1).

The results revealed that for the low-low, low–high and high-low participants, there was a significant positive correlation between stress exposure and level of depression (B = 1.97, CI 95% [1.41, 2.54], *t* = 6.84*, p* < 0.001; B = 1.03, CI 95% [0.36, 1.71], *t* = 3.01, *p* = 0.003; B = 1.20, CI 95% [0.57, 1.84], *t* = 3.71,* p* < 0.001, respectively), indicating that for these educators higher stress exposure was associated with higher levels of depressive symptoms. However, for the high-high participants, no significant correlations were found between stress exposure and level of depression (B = 0.26, CI 95% [− 0.34, 0.87], *t* = 0.86, *p* = 0.387).

## Discussion

Educators are frequently exposed to school-related stress that may impair their functioning and well-being^14,40,52^. The current study aimed to test the effect of internal (regulatory flexibility) and external (school climate) resources on the relationship between school-related stress and the tendency to develop depressive symptoms in this population. The results revealed that educators with high regulatory flexibility and a positive school climate exhibited lower depressive symptoms independent of their school-related stress exposure. On the other hand, for individuals with either low regulatory flexibility and/or poor school climate, increased school-related stress exposure was associated with greater depressive severity. These results are in accordance with the COR model, suggesting that the loss of internal and external resources is associated with mental health deterioration^6,49,53^.

The protective effect of regulatory flexibility adds to a growing body of research showing that regulatory flexibility moderates the relationship between exposure to aversive events and clinical symptoms in various populations, including active-duty first responders^38,40,46^. For example, active-duty firefighters with increased regulatory flexibility maintained lower PTSD symptoms across their service, whereas those with reduced regulatory flexibility showed increased symptoms across time and trauma exposure. The effect of a positive school climate supports and extends previous findings, which show that a positive school climate benefits educators' well-being^46,54^.

However, the most innovative findings of the present study are the dependency between regulatory flexibility and school climate. Specifically, the results suggest that only for educators with both high regulatory flexibility and a positive school climate, depression symptom severity remains low independent of exposure to school-related stress. Hence, only their cumulative effect mitigates negative stress outcomes. The results align with the fundamental principles of the COR model, which underscore the importance of examining internal and external resources as distinct constructs while also considering potential interactions between these resource types^29^. It offers potential support for the distinct contribution of diverse resources^55^ yet suggests that combining these resources may be necessary to withstand the detrimental effects of stress effectively. Finally, it further supports the perspective of the self as shaped within a sociocultural context, highlighting the interconnectedness between the self and its surrounding environment^6^.

While important, the current study may have several limitations. First, most of the sample is women. Previous studies suggested that women experience stressful situations differently than men^56^ and have a higher tendency to develop depressive symptoms and mental deterioration^57^. Hence, it is possible that the levels of symptoms measured in this sample increased due to the majority of women. However, gender distribution in the current sample is consistent with the high percentage of women among educators (82%, according to the Israeli National Central Bureau of Statistics, 2020). Moreover, gender was included as a control variable in the moderation model. Hence, the results are above and beyond its possible effects. With that being said, a future study may aim to test a larger number of male educators, possibly in countries where the educational profession has a larger male representation. Finally, as seen in Table 1, the sample is very varied and includes educators from different school levels with various roles. Hence, the generalizability of the results is relatively high. Second, while many studies have focused on depression and show that this is the most common psychopathology in educators, future studies may aim to include positive outcomes as well. For example, it was found that improving school climate strengthens school satisfaction^58^ as well as resilience and wellness^59^. Future studies may test whether regulatory flexibility and school climate moderate the relationship between stress exposure and these variables. Finally, the current study examined a particular type of flexibility. Recently, research has focused on the impact of different types of flexibility and their distinct effects^37^. While the current study has focused on a promising, highly relevant type of flexibility^45^, further research is needed to examine the implications of other types of flexibility, such as cognitive and psychological flexibility.

The results of the current study add to growing evidence that calls for attention to the well-being of educators^55,60,61^ and to developing targeted interventions. This is especially important given the possible vicious cycle described by the COR model, suggesting that limited resources under conditions of continual exposure to stress lead to further resource depletion^33^. Previous studies have shown the effectiveness of various interventions. For instance, it was found that using mindfulness has effectively enhanced teacher well-being, improved the school climate, and supported short-term reductions in teacher burnout^62^. Moreover, it was suggested that interventions that aim to improve mentalization by deepening the understanding and reflection on mental states might facilitate the ability of educators to cope with stressful situations^63^. The current study suggests that new interventions should aim to improve both external resources, specifically school climate (for example, by improving the interpersonal relationships between the school educators and refining the organizational structures) and internal resources, specifically enhance the way educators choose and apply regulatory strategies that suit the environmental demands.

In summary, the present study demonstrated that in line with the COR model, both regulatory flexibility and school climate, representing distinct internal and external resources, play a crucial role in mitigating the adverse effects of school-related stress exposure in educators. These findings hold potential for developing targeted interventions aimed at reducing depressive symptoms and enhancing well-being among educators. This is of particular significance considering the direct influence of educators' well-being on their capacity to function effectively and guide their students toward personal and academic growth.

## Data Availability

The datasets generated and analyzed during the current study are available in the OFS repository, https://osf.io/g8acw?view_only=05627a70bb5a4db78643bf28c1c3110f.
